# Body mass index and glioma risk: A prospective multicenter study

**DOI:** 10.3389/fendo.2022.933921

**Published:** 2022-08-29

**Authors:** Chuan Shao, Hui Tang, Xiaoya Wang, Jiaquan He, Pan Wang, Nan Wu

**Affiliations:** ^1^ Department of Neurosurgery, Chongqing General Hospital, Chongqing, China; ^2^ Department of Neurosurgery, Nanchong Central Hospital, The Second Clinical Medical College, North Sichuan Medical College, Nanchong, China; ^3^ Graduate Institute, Chongqing Medical University, Chongqing, China

**Keywords:** body mass index, glioma, cohort, obese, overweight, obesity

## Abstract

**Background:**

The association between glioma risk and body mass index (BMI) remains obscure.

**Methods:**

This study aimed to assess the association between glioma risk and BMI in the Prostate, Lung, Colorectal, and Ovarian (PLCO) Cancer Screening Trial. Cox proportional hazards regression was used to calculate the hazard ratios (HRs) and 95% confidence intervals (CIs).

**Results:**

The onset of a total of 269 gliomas was observed during a median follow-up period of 12.04 years. Compared with the normal weight, overweight (HR: 1.05; 95% CI: 0.80, 1.39) and obesity (HR: 0.91; 95% CI: 0.56, 1.39) were not significantly associated with glioma risk. Further analysis showed a nonlinear relationship between glioma risk and BMI in men but not women. The multivariable-adjusted HRs per unit increase in BMI were 0.94 (95% CI: 0.89, 1.00; *P* = 0.037) in men with BMI >25 kg/m^2^ and 1.16 (95% CI: 0.98, 1.38; *P* = 0.075) in men with BMI <25 kg/m^2^.

**Conclusion:**

The present data provide evidence that there may be a nonlinear association between BMI and glioma risk in men. The risk of glioma decreased with increasing BMI among men with BMI >25 kg/m^2^. Future studies are needed to validate our observation.

## Introduction

Glioma, a malignancy for which very few well-established environmental risk factors have been identified except for high doses of ionizing radiation, represents approximately 80.9% of malignant brain tumors (1, 2). Currently, patients diagnosed with glioma have minor benefits in survival time, despite the availability of multimodal optimal therapies, including surgery, chemotherapy, and radiation (1, 3). In particular, glioblastoma, accounting for 49.1% of malignant brain tumors, has the lowest median survival (8 months) and a 5-year relative survival of 5% (1).

The prevalence of overweight and obesity and the associated burden have risen considerably worldwide in recent decades. Between 1975 and 2016, the worldwide prevalence of obesity increased from 7% to 16% in women and from 3% to 12% in men (4). Correspondingly, approximately 40% of adults have excessive body weight, defined as a body mass index (BMI) of ≥25 kg/m^2^ (4). The rapidly increasing trend of excess body weight has taken a major toll on global health. According to the Global Burden of Disease study data published in 2017, excess body weight accounted for as many as 120 million disability-adjusted life years and 4.0 million deaths worldwide (5). Regarding cancer burden, another report has indicated that excessive body weight is present in approximately 3.9% of all cancer cases (6).

Epidemiological evidence with some consistency suggested a causal link between body fatness and some cancer risk, including multiple myeloma, meningioma, and cancers of thyroid, esophagus (adenocarcinoma), breast (postmenopausal), stomach (cardia), colon and rectum, liver, pancreas, gallbladder, endometrium, kidney, and ovaries (4, 7). However, evidence of the association between BMI and the risk of glioma is limited and somewhat inconsistent (8–17). Most studies have shown no association between overweight or obesity and glioma, whereas three studies have reported that obesity earlier in life (at ages 18 and 21) or at baseline is associated with an increased risk of glioma (8, 10, 12, 17). The disparity in these findings may be associated with aspects of study design, such as the sample size, study participants, exposure measurement, confounders, and study type. To further shed light on the relationship between BMI and glioma risk, we evaluated the data on self-reported body weight and height in the Prostate, Lung, Colorectal, and Ovarian (PLCO) Cancer Screening Trial.

## Materials and methods

### Data source

The PLCO Cancer Screening Trial, which recruited 154,887 men and women aged 42–78 years in 1993–2001, was a multicenter prospective study in the United States. The detailed design and implementation of PLCO have been described elsewhere (18). Ethical approval was obtained from all participating centers in the PLCO trial before study activation. The trial was initially designed to evaluate whether screening exams might decrease mortality from prostate, lung, colorectal, and ovarian cancers and was performed in 10 centers: Alabama; Missouri; Hawaii; Pennsylvania; Minnesota; Colorado; Wisconsin; Washington, DC; Michigan; and Utah. Data on all cancer diagnoses and the specific causes of death were also collected.

### Data collection and participant selection

At the start of the study, all participants were asked to complete a baseline questionnaire involving the demographics, medical history, anthropometric factors (i.e., height and weight), smoking, sex-associated exposures (i.e., exogenous hormone use, menstrual and reproductive factors for women, and prostate-associated factors for men), and other risk factors. Approximately 97% of the questionnaires were completed (n = 149,969). We subsequently excluded 9,699 participants for having a history or an undetermined history of cancer (n = 6,941) or unavailable data regarding follow-up time (n = 570), height data (n = 1,162), and body weight data (n = 1,026). Ultimately, 140,270 participants were finally included (**Supplementary Figure S1**).

### Case ascertainment

Certification of glioma diagnoswas performed *via* an annual study questionnaire or National Death Index. Study participants, spouses, or other proxies were asked to disclose whether a cancer was diagnosed. When a diagnosis of cancer was reported, questions regarding the cancer type, diagnosis date, the address of hospital or clinic, and the name, address, and contact number of the physician were required to be answered. For every undetermined cancer, medical records on the cancer site and morphology [International Classification of Diseases for Oncology Second Edition ICD-O-2 codes] were abstracted. Follow-up lasted from trial entry to glioma diagnosis, death, withdrawal from the study, or trial censoring (31 December 2009).

### Exposure data

Body weight and height for all participants at baseline and 20 and 50 years of age were retrospectively collected in the baseline questionnaire. Participants were excluded if they had a weight below 60 lb or had a height below 48 inches or above 78 inches for women or 84 inches for men. BMI was calculated as weight in kg/height in m^2^. Moreover, participants with a BMI <15 kg/m^2^ were also excluded according to the predefined study design.

### Statistical analyses

Cox proportional hazards regression analyses were adopted to calculate the hazard ratios (HRs) and their corresponding 95% confidence intervals (CIs). Tests for proportional hazard assumptions revealed no departures from proportionality (*P* > 0.05). Multivariable risk estimates were adjusted for age (smooth) at recruitment (**Supplementary Figure S2**), sex (men vs. women), race (white, non-Hispanic vs. other/unknown), marital status (ever married or living as married vs. never married), education (up to post high school vs. at least some college), smoking status (never vs. former vs. current smoking), and height. For women, the risk estimates were additionally adjusted for oral contraceptive (OC) use (never vs. ever use) and hormone replacement therapy (HRT; never vs. ever use). BMI was initially assessed according to WHO categories, and tests for linear trend were performed by modeling the median value of each BMI category as continuous variables. Owing to the small numbers of underweight participants (<18.5 kg/m^2^; n = 1,044), normal weight was combined with underweight as the reference category (<25 kg/m^2^); thus, BMI was classified into three categories: normal weight (<25 kg/m^2^), overweight (25–30 kg/m^2^), and obese (≥30 kg/m^2^). To address potential reverse causality, sensitivity analysis was limited to participants with more than 2 years of follow-up. To assess the possible effects of sex differences, a subgroup analysis by sex was performed. Then, we used restricted cubic spline functions with four default knots to model a smooth curve (19, 20), which was used to assess the relationship between glioma events and BMI and was fitted using the “spline smoothing plot module” in Empower software. To test for possible non-linearity, a log-likelihood ratio test comparing the one-line linear model (Model I) to segmented regression model (Model II) was performed (21). All statistical analyses were performed in R software (version 3.4.3; http://www.R-project.org) and Empower (version 2.0; X&Y Solutions, Inc., Boston, MA, USA).

## Results

During a median follow-up of 12.04 years, 170 men and 99 women developed incident glioma. The baseline characteristics according to the BMI categories were shown in **Table 1**. Most participants bore excess weight, particularly men, and participants with higher BMIs tended to be young and not be current smokers.

**Table 1:** Basic characteristic according to body mass index in the PLCO study.

Characteristic^a^	Body mass index (kg/m^2^)			*P*-value
	<25 (n=47,659)	≥25, <30 (n=59,545)	≥30 (n=33,066)	
Age (y); median (Q1-Q3)	62.00 (58.00-67.00)	62.00 (58.00-67.00)	61.00 (57.00-66.00)	<0.001
Height (inches); median (Q1-Q3)	66.00 (64.00-69.00)	68.00 (65.00-71.00)	67.00 (64.00-70.00)	<0.001
Arm; N (%)				0.104
Intervention	24,055 (50.47%)	30,043 (50.45%)	16,907 (51.13%)	
Control	23,604 (49.53%)	29,502 (49.55%)	16,159 (48.87%)	
Sex; N (%)				<0.001
Men	19,013 (39.89%)	35,577 (59.75%)	16,055 (48.55%)	
Women	28,646 (60.11%)	23,968 (40.25%)	17,011 (51.45%)	
Race; N (%)				<0.001
White, Non-Hispanic	41,979 (88.08%)	53,132 (89.23%)	28,949 (87.55%)	
Other/unknown	5,680 (11.92%)	6,413 (10.77%)	4,117 (12.45%)	
Education; N (%)				<0.001
Up to post high school	18,276 (38.42%)	25,535 (42.97%)	16,206 (49.11%)	
At least some college	29,287 (61.58%)	33,897 (57.03%)	16,795 (50.89%)	
Marital status; N (%)				<0.001
Ever married or living as married	45,804 (96.26%)	57,720 (97.10%)	31,789 (96.32%)	
Never married	1,782 (3.74%)	1,724 (2.90%)	1,216 (3.68%)	
Smoking; N (%)				<0.001
Never	23,249 (48.79%)	26,688 (44.83%)	14,909 (45.09%)	
Current	6,445 (13.52%)	5,802 (9.75%)	2,665 (8.06%)	
Former	17,960 (37.69%)	27,047 (45.43%)	15,488 (46.85%)	
OC (women); N (%)				<0.001
Never	12,855 (44.91%)	11,146 (46.54%)	7,699 (45.30%)	
Ever	15,769 (55.09%)	12,803 (53.46%)	9,296 (54.70%)	
HRT (women); N (%)				<0.001
Never	8,132 (28.51%)	7,809 (32.74%)	6,721 (39.75%)	
Ever	20,396 (71.49%)	16,040 (67.26%)	10,187 (60.25%)	

PLCO, prostate, lung, colorectal, and ovarian; BMI, body mass index; N, number; y, years; OC, oral contraceptive; HRT, hormone replacement therapy.

a There were 274, 235, 623, 17, 57, and 275 subjects missing the data for education, marital status, smoking, OC, and HRT, respectively.

No significant associations were observed in the analyses of BMI at baseline. With the normal BMI group as the reference category, the multivariable-adjusted HR was 1.05 (95% CI: 0.80, 1.39) for overweight and 0.91 (95% CI: 0.64, 1.29) for obesity. Our sensitivity results remained unchanged when the analysis was limited to participants with more than 2 years of follow-up. The multivariable-adjusted HR was 1.08 (95% CI: 0.80, 1.46) for overweight and 0.99 (95% CI: 0.69, 1.43) for obesity. In subgroup analysis by sex, no significant differences in overall results were identified (**Table 2**).

**Table 2:** HRs for the association between glioma risk and BMI at baseline.

Exposure	Cases	Cohort	Non-adjusted model		Adjusted model	
			HR (95% CI)	*P*	HR (95% CI)	*P*
BMI in all participants						
<25 kg/m^2^	89	47,659	1.0		1.0	
≥25, <30 kg/m^2^	127	59,545	1.15 (0.88, 1.51)	0.310	1.05 (0.80, 1.39)	0.724
≥30 kg/m^2^	53	33,066	0.91 (0.64, 1.27)	0.570	0.91 (0.64, 1.29)	0.594
*P* for trend				0.626		0.616
BMI in men						
<25 kg/m^2^	48	19,013	1.0		1.0	
≥25, <30 kg/m^2^	90	35,577	1.00 (0.71, 1.42)	0.992	1.02 (0.72, 1.46)	0.901
≥30 kg/m^2^	32	16,055	0.83 (0.53, 1.29)	0.407	0.88 (0.56, 1.39)	0.583
*P* for trend				0.408		0.587
BMI in women						
<25 kg/m^2^	41	28,646	1.0		1.0	
≥25, <30 kg/m^2^	37	23,968	1.10 (0.70, 1.71)	0.689	1.10 (0.71, 1.73)	0.662
≥30 kg/m^2^	21	17,011	0.92 (0.54, 1.55)	0.744	0.97 (0.57, 1.66)	0.921
*P* for trend				0.787		0.965

BMI, body mass index; HR, hazard ratio; 95% CI, 95% confidence interval; OC, oral contraceptive; HRT, hormone replacement therapy.

Adjusted for age (smooth), sex, education, race, marital status, smoking status, and height. For women, the risk estimates were additionally adjusted for OC and HRT.

**Figure 1:**
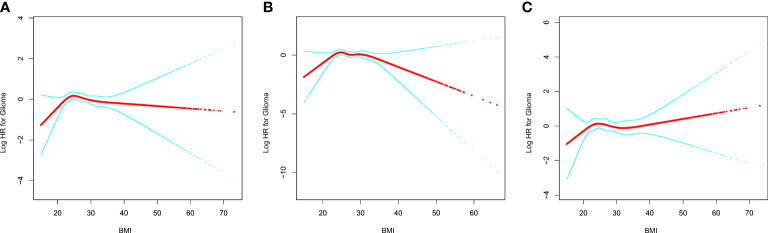
The relationship between glioma risk and BMI. **(A)** All participants; **(B)** men; **(C)** women. Adjusted for age (smooth), sex, race, marital status, education, smoking, and height. For women, the risk estimates were additionally adjusted for OC (never vs. ever use) and HRT (never vs. ever use). BMI, body mass index; OC, oral contraceptive; HRT, hormone replacement therapy.

In **Figure 1**, we used restricted cubic splines to visualize the association between BMI and glioma risk. The risk of glioma increased until a BMI of approximately 25 kg/m^2^, then started to decrease (**Figure 1**). Above 25 kg/m^2^, the multivariable-adjusted HR per unit increase in BMI was 0.96 (95% CI: 0.93, 1.00; *P* = 0.074). Below 25 kg/m^2^, the multivariable-adjusted HR per unit increase in BMI was 1.11 (95% CI: 1.00, 1.23; *P* = 0.055). Further subgroup analysis showed that a nonlinear relationship between glioma risk and BMI at baseline was only identified in men, whereas a linear relationship was observed in women (**Figure 1**, **Table 3**). The multivariable-adjusted HR per unit increase in BMI was 0.94 (95% CI: 0.89, 1.00; *P* = 0.037) in men with BMI >25 kg/m^2^. Correspondingly, the multivariable-adjusted HR per unit increase in BMI was 1.16 (95% CI: 0.98, 1.38; *P* = 0.075) in men with BMI <25 kg/m^2^.

**Table 3:** One-line linear regression or segmented line regression results for the relationship between glioma risk and BMI.

Model	Men	Women	All
	Adjusted HR (95% CI) *P*-value	Adjusted HR (95% CI) *P*-value	Adjusted HR (95% CI) *P*-value
Model I			
One-line slope	0.980 (0.942, 1.020) 0.321	1.006 (0.970, 1.044) 0.740	0.993 (0.966, 1.020) 0.598
Model II			
Turning point	25 kg/m^2^	25 kg/m^2^	25 kg/m^2^
<25 slope 1	1.165 (0.985, 1.378) 0.075	1.066 (0.931, 1.220) 0.355	1.108 (0.998, 1.231) 0.055
>25 slope 2	0.942 (0.891, 0.997) 0.037	0.991 (0.941, 1.044) 0.734	0.965 (0.929, 1.003) 0.074
*P* for LRT test	0.024	0.373	0.026

BMI, body mass index; HR, hazard ratio; 95% CI, 95% confidence interval; OC, oral contraceptive; HRT, hormone replacement therapy.

Data were presented as adjusted HR (95% CI) P-value; Model I, one-line linear regression analysis; Model II, segmented regression model. LRT test, Logarithmic likelihood ratio test (P-value <0.05 means Model II is significantly different from Model I, which indicates a nonlinear relationship); adjusted for age (smooth), sex, education, race, marital status, smoking status, and height. When subgroup analysis was performed by sex, the risk estimates were additionally adjusted for OC and HRT.

## Discussion

In this prospective cohort study including 140,270 men and women 42–78 years of age in the United States, we found that there may be a nonlinear relationship between glioma risk and BMI in men and a turning point (25 kg/m^2^) was indicated.

Mixed findings on the association between early adult body weight and glioma risk have been reported (10–13, 16, 17). In a study of 499,437 participants with 8.2 years of follow-up, reporting BMI at baseline and 18, 35, and 55 years of age, Moore et al. (10) found that being underweight at 18 years of age was associated with a lower risk of glioma, whereas excess body weight was associated with a higher risk of glioma. However, the only significant association was detected for obesity [risk ratio (RR) 3.91; 95% CI: 2.08, 7.35]. In a further analysis of BMI at baseline and 35 and 55 years of age, no meaningful link between BMI and glioma risk was found (10). In the European Prospective Investigation Into Cancer and Nutrition study, Michaud et al. (11) have analyzed data on BMI at age 20 in a subset cohort of 127,494 women and 73,834 men and have found no association between excess body weight at 20 years of age and subsequent risk of glioma. In a United States-based case–control study, a 33% reduced risk in glioma was observed among participants with a BMI <18.5 kg/m^2^ at age 21 years compared to those with normal BMI (12). Interestingly, greater BMI was associated with a slightly increased risk of glioma, with an RR of 1.04 (95% CI: 1.02, 1.07) per 1 kg/m^2^ increase when BMI at 21 years was modeled as a continuous variable (12). However, no trend of increased risk of glioma was identified for BMI at baseline (defined as 1–5 years before interview) (12). Findings from pooled results of the Health Professionals Follow-Up Study and the Nurses’ Health Study showed that higher BMI at age 18 in women and at age 21 in men was associated with an increased risk of glioma (HR: 1.37; 95% CI: 1.02, 1.85 comparing >25 to <25 kg/m^2^) (13). In the Women’s Health Initiative (16), none of the risk estimates were significant at ages 18, 35, and 50. These findings suggested that early-life exposure and energy balance-related factors may be responsible for glioma development, and thus this issue remains to be further elucidated.

The relationship between waist-to-hip ratio (WHR) and glioma risk has gained attention in recent years. A multicenter prospective cohort study of 380,775 participants in 10 European countries has indicated that greater WHR is associated with a non-significantly decreased risk of glioma, with an HR of 0.92 (95% CI: 0.66, 1.28) for the highest vs. lowest tertile (11). In contrast, higher WHR is associated with a modestly but non-significantly increased risk of glioma in a prospective cohort study of 161,119 postmenopausal women (HR: 1.34; 95% CI: 0.90, 2.00) (16). Moreover, a nationwide study of 6,833,744 Koreans with a median follow-up time of 7.30 years has indicated that abdominal obesity (defined as waist circumference ≥90 cm for men and 85 cm for women) is significantly associated with glioma risk, and the relationship was more pronounced in abdominal obesity with BMI >25 kg/m^2^ (8). Compared with BMI, WHR is a more accurate surrogate marker for obesity because it measures the anatomic distribution of body fat and distinguishes between lean muscle mass and fat mass (22). Therefore, identification of the relationship between glioma risk and general and abdominal obesity is warranted in future studies.

Our subgroup results provided some evidence of a nonlinear association in men, although some results were borderline significant. Potential explanations for the sex discrepancies may be associated with the roles of sex hormones in cancer pathogenesis. Adiposity is positively associated with estrogen concentration and negatively associated with testosterone concentration in men but positively associated with testosterone concentration in women (23). Some evidence indicates that testosterone regulates cell metabolism; affects various enzymes and signaling pathways that enhance energy balance; and improves the uptake, utilization, and storage of lipids and glucose (24). The expression of steroid hormone receptors and the proliferation of glioma cells inhibited by estrogens have been demonstrated through *in vitro* studies (25, 26). Furthermore, estrogen receptor (ERβ) overexpression restrains cell proliferation, neurosphere formation, and the self-renewal ability of glioma stem cells; induces apoptosis; and decreases stemness marker expression (27). Additional epidemiology findings indicating that women have a 40%–50% lower incidence of glioma than men (28) have added support to the strength of the sex differences. Together, these findings indicate that obese men may have a lower risk of glioma.

Currently, the biological mechanisms appear to be complicated or even to show a paradoxical association between glioma and obesity. Adipose tissue is an endocrine organ, and increased adipose tissue results in secretion of higher levels of insulin-like growth factor (IGF), leptin, inflammatory cytokines, and female hormones and lower levels of adiponectin (29). The IGF signaling pathway has been indicated to promote proliferation, growth, migration, and invasion of glioma cells, as discussed comprehensively in a previous review (30). Moreover, IGF-I and -II, their receptors, and several IGF-binding proteins (IGFBP-2, -3, -4, -5) are overexpressed in glioblastoma compared with normal brain tissue or low-grade glioma; this overexpression is associated with poor prognosis or less favorable responses to therapy (30). Similarly, leptin enhances the production of matrix metalloproteinase (MMP)-13 through the p38-mitogen activated protein (MAP) kinase and nuclear factor (NF)-κB pathway, thus promoting the migration and invasion of C6 glioma cells (31). Moreover, adiponectin (Acrp30) treatment prevents DNA synthesis and cell proliferation in U87 and U251 glioma cell lines, thus resulting in arrest in the G1 phase of the cell cycle (32). AdipoR2, one of two Acrp30 receptors, is less expressed in high-grade glioma than low-grade glioma or normal brain tissue; higher expression of AdipoR2 is associated with a favorable prognosis (33). Further biological experiments have shown that AdipoR2 overexpression inhibits glioma cell proliferation through the adenosine monophosphate associated protein kinase (AMPK)–mammalian Target of Rapamycin (mTOR) signaling pathway (33). Female sex hormones have been found to have a protective effect against glioma, as described above (25–27). Recently, Almeida et al. (34) have developed an *in vitro* rodent model to assess the effects of adipocyte-released factors on glioma biology. The findings have indicated that adipokines restrain or promote the growth and progression of glioma cells in different phases (34). Together, the biological mechanisms linking obesity and glioma development remain a topic of ongoing research.

The major merits of this study include its prospective design with a long follow-up period and large sample size. However, three major limitations should be acknowledged. First, a small number of cases of glioma were identified in PLCO, and thus the results might possibly have been due to chance. Second, the measurement of height and weight relied on self-reported baseline questionnaires. Additionally, the changes in weight during the follow-up period were not considered in our study, and misclassification might potentially have occurred, thus masking the true associations of BMI with glioma risk. Another is that residual or unmeasured confounding was a major concern owing to the nature of the observational design.

In summary, this prospective cohort study provides evidence that there may be a nonlinear association between BMI and glioma risk in men. The risk of glioma decreased with increasing BMI among men with BMI >25 kg/m^2^. Further studies are needed to confirm this finding.

## Data availability statement

The data of the current study are available from NIH PLCO study group but restrictions apply to the availability of these data, which were used under license for the current study. Any requests to access the datasets should be directed to https://cdas.cancer.gov/plco/.

## Ethics statement

Ethical approval was obtained from all participatin centers (Alabama, Missouri, Hawaii, Pennsylvania, Minnesota, Colorado, Wisconsin, Washington DC, Michigan, and Utah) in the PLCO trial before study activation. The patients/participants provided their written informed consent to participate in PLCO trial.

## Author contributions

CS, HT, and XW contributed to conception and design of the study. CS, HT, XW, and PW data cleaned and statistical analyzed. CS, HT, XW, PW, NW, and JH wrote the first draft of the manuscript. All authors contributed to manuscript revision, read, and approved the submitted version.

## Acknowledgments

The authors thank the National Cancer Institute for access to NCI's data collected by the Prostate, Lung, Colorectal and Ovarian (PLCO) Cancer Screening Trial. The statements contained herein are solely those of the author and do not represent or imply concurrence or endorsement by NCI.

## Conflict of interest

The authors declare that the research was conducted in the absence of any commercial or financial relationships that could be construed as a potential conflict of interest.

## Publisher’s note

All claims expressed in this article are solely those of the authors and do not necessarily represent those of their affiliated organizations, or those of the publisher, the editors and the reviewers. Any product that may be evaluated in this article, or claim that may be made by its manufacturer, is not guaranteed or endorsed by the publisher.

## Author disclaimer

The statements contained herein are solely those of the author and do not represent or imply concurrence or endorsement by NCI.
