# Supplementary material for: Body mass index and glioma risk: A prospective multicenter study
Source: Front Endocrinol (Lausanne). 2022 Aug 29;13:933921. doi: 10.3389/fendo.2022.933921 (PMC9465449; doi:10.3389/fendo.2022.933921)
Supplement: Supplementary file 1 [file DataSheet_1.docx]

Supplementary Material


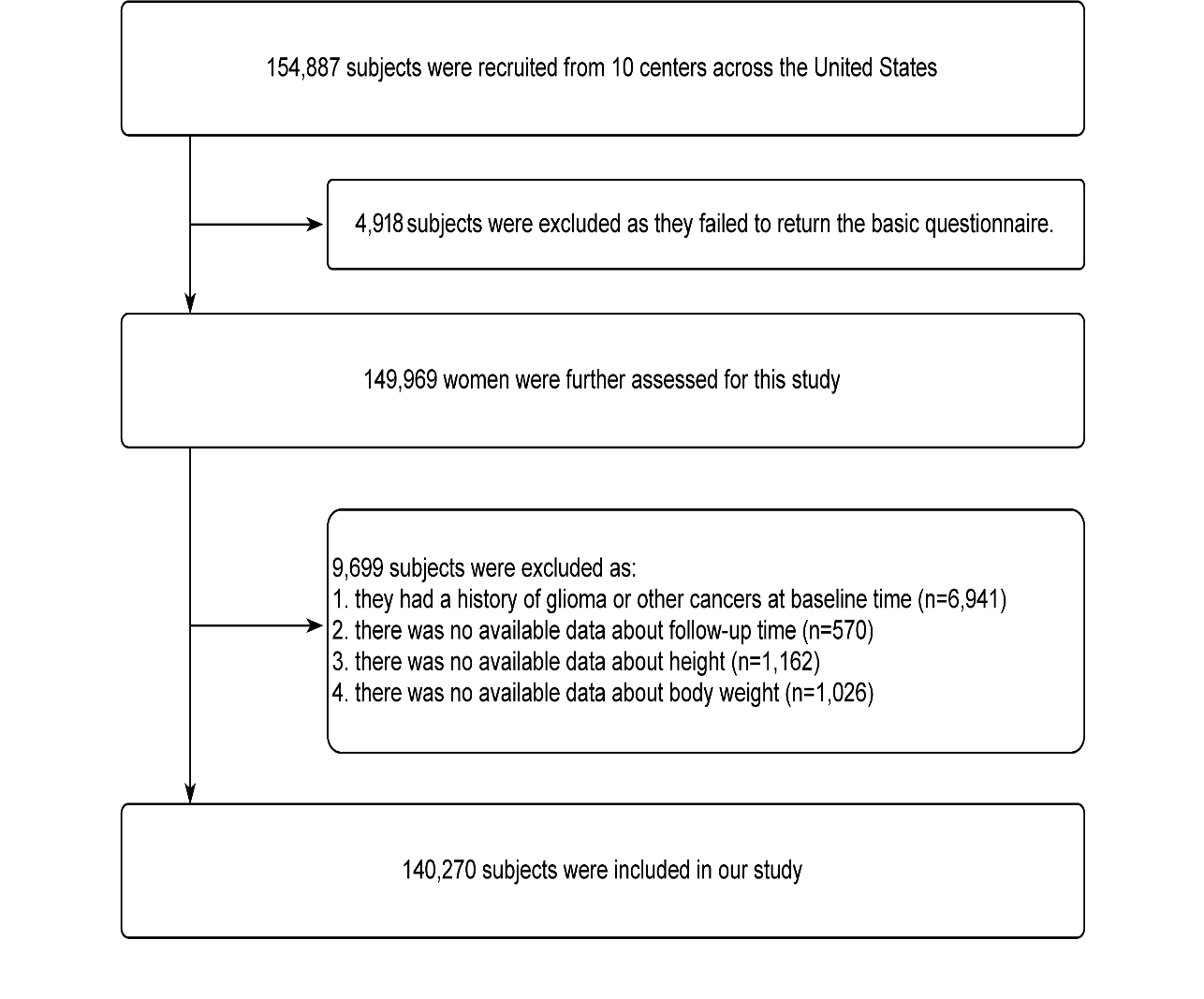


**Supplementary Figure 1**. The study flowchart of identifying subjects.


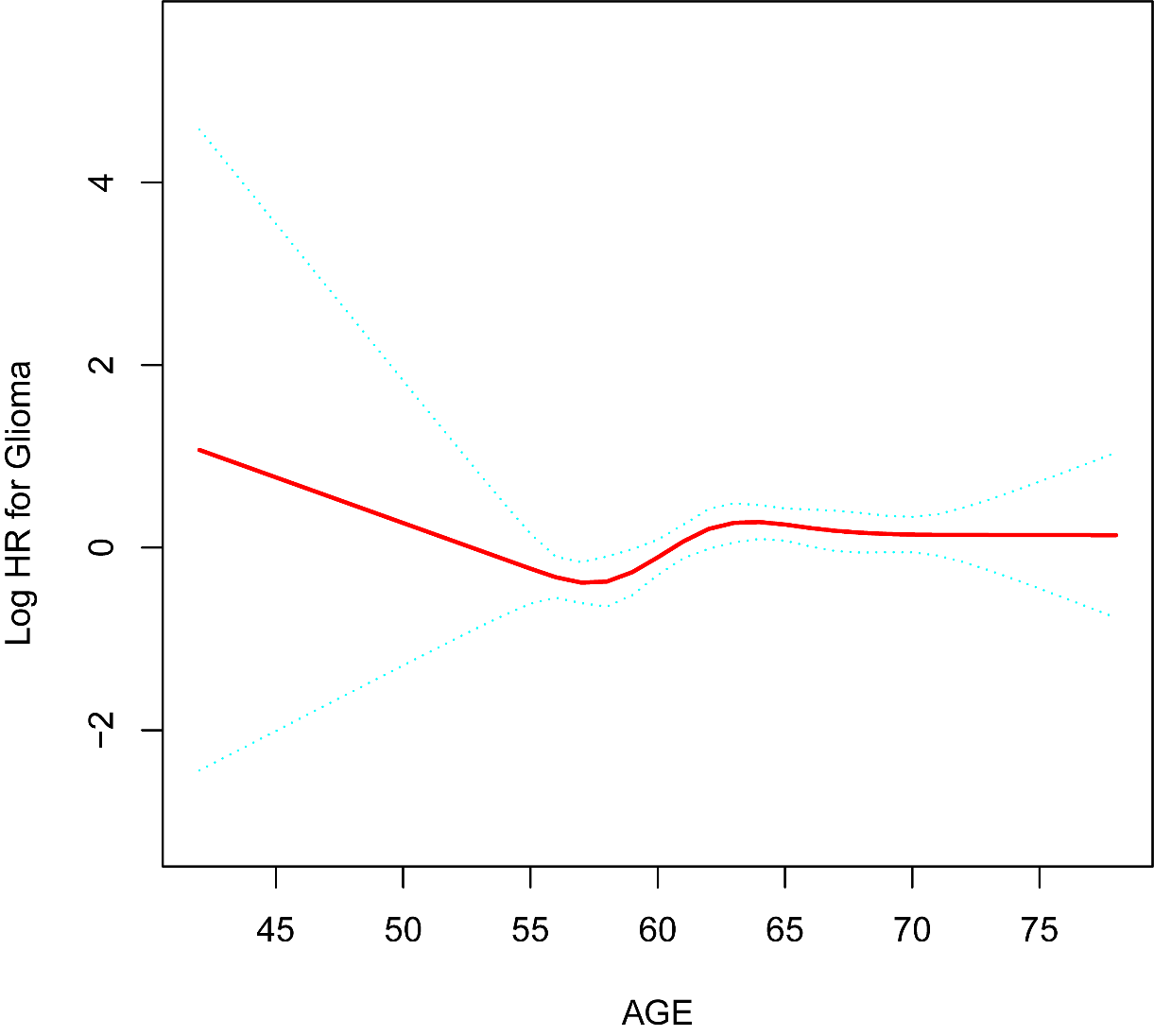


**Supplementary Figure 2.** The relationship between glioma risk and age.
